# Hydrochemical Evolution Mechanisms of Shallow Groundwater and Its Quality Assessment in the Estuarine Coastal Zone: A Case Study of Qidong, China

**DOI:** 10.3390/ijerph17103382

**Published:** 2020-05-12

**Authors:** Yao Wen, Jiahao Qiu, Si Cheng, Changchang Xu, Xiaojiang Gao

**Affiliations:** Department of Environmental Science and Engineering, Fudan University, Shanghai 200433, China; 17210740015@fudan.edu.cn (Y.W.); wxthhpzl@126.com (J.Q.); 18210740038@fudan.edu.cn (S.C.); 19210740063@fudan.edu.cn (C.X.)

**Keywords:** hydrochemistry, shallow groundwater, coastal zone, Yangtze estuary, groundwater quality

## Abstract

Identification of geochemical characters and the influence of river and seawater on coastal areas are of significant impact on water resources management in coastal areas globally. Thus, it requires careful investigation of the hydrochemical evolution mechanisms and assessment of the quality of shallow groundwater. The study specifically focused on the estuarine coastal zone of Qidong, China, a city located on the Yangtze estuary. Thirty shallow groundwater samples and five surface water samples were collected during a field investigation, and 25 water quality indexes were analyzed. Methods including mathematical statistics, Gibbs figure, Piper diagram, and ionic rations were used to analyze the hydrochemical characteristics and evolution mechanisms. The spatial distribution of key parameters was assessed using a GIS-based spatial gridding technique. Results showed that the shallow groundwater in this estuarine coastal zone is weakly alkaline. The major hydrochemical parameters, including total dissolved solids (TDS), Na^+^ and Cl^−^, Mg^2+^, and SO_4_^2−^, displayed similar spatial distributions; the distributions of Ca^2+^ and Mg^2+^ were irregular; the distributions of SO_4_^2−^ and HCO_3_^−^ shared a similar trend, increasing gradually from the southern to northern regions of the study zone; and the values of NO_3_^−^ and NH_4_^+^ were generally low in the study area. The hydrochemistry of the groundwater consists of HCO_3_-CaMg type and HCO_3_-Na, with HCO_3_·Cl-Na type and Cl-Na being the dominant constituents toward the coastal strip. The coastal aquifers are subjected to the ongoing influence of seawater intrusion, ion exchange processes, freshwater infiltration, and mineral (carbonate and silicate) dissolution, which impact groundwater evolution. Most of the groundwater samples are unsuitable for drinking purposes, but more than 90% of samples have irrigation suitability, based on the WHO standards and the classifications of sodium absorption ratio (SAR), soluble-sodium percentage (SSP).

## 1. Introduction

In general, estuarine coastal zones are a hotspot for economic development and trade, industrial activity, and population aggregation. Additionally, groundwater is a crucial resource for agricultural, industrial, and domestic output, and it has become one of the most significant factors restricting sustainable regional socio-economic development and ecological environmental protection. Groundwater utilization in the coastal provinces of China reached 4.4 × 10^10^ m^3^ in 2009 [1]. Coastal areas are often the final discharge zones for regional groundwater flow systems, with the continual mixing of coastal groundwater and seawater [2]. However, with ongoing economic development and high-intensity human activity, estuarine coastal zones are subject to significant environment damage, the equilibrium between freshwater and seawater has been compromised, causing many environmental issues, such as water resources pollution, water salinization, groundwater depth decline, declining fresh and marine water quality, seawater intrusion, land degradation, and wetland destruction [3,4,5].

The direct link between the groundwater in coastal aquifers and seawater presents a unique challenge to groundwater sustainability in coastal regions and has become a primary restraint in groundwater utilization [6]. Groundwater hydrochemistry provides an indication of the chemical composition of groundwater, revealing the nature of groundwater circulation pathways and the entire water flow system. Therefore, a fundamental body of work has focused on investigating the chemical characteristics of coastal groundwater and the distribution of different types of water chemistries [7,8,9,10]. There are differences in distribution of lithology, velocity of groundwater flow, geological factors, and recharge water in the groundwater aquifer following the path of recharge area-runoff area-discharge area. The hydrochemical characteristics and evolution of groundwater are influenced by the provenance of lithostratigraphy and sedimentary environment, hydrogeological conditions and runoff factors, as well as external conditions such as climate change, saline-water encroachment, and anthropogenic activities. Up until now, scholars have primarily studied the chemical characteristics of groundwater and the formation and evolution of hydrochemical composition by using such technical methods as mathematical statistics and geo-statistics, the Piper diagram, the ion proportional coefficient, the mineral saturation index, isotope tracers, and hydrogeochemical simulation [11,12,13,14,15,16,17,18]. The Gibbs diagram has also been applied that provides insightful guidance on the formation mechanisms of the hydrochemistry of groundwater [3,19,20]. In particular, the salinization of coastal groundwater has received widespread attention [21,22,23], while the distribution of nutrient salts in the groundwater of coastal regions has also attracted some research interest [24,25]. The geo-spatial analysis based on the geospatial mapping techniques as an intuitionistic and convenient approach can yield significant information regarding the origin of groundwater components and the evolution of groundwater salinization [26,27,28,29]. These conditions impose significant management challenges and there is a need for effective means and policies to protect the shallow groundwater which is closely related to human life and health.

The Yangtze estuary coastal area has a flat terrain and a large tidal range and is subject to a water dilution process from the Yangtze River, as well as seawater intrusion. The unique geographical location of it is helpful to expand the research field of coastal zone hydrochemistry worldwide and provide reference for similar situations. In addition, it is of great consequence to study the environmental geochemistry of groundwater in coastal areas in order to further understand the environmental evolution of regional groundwater [30]. Therefore, in order to elucidate the interaction mechanisms between coastal groundwater and the environment, it is crucial to study the spatial distribution and evolution rules of coastal groundwater hydrochemistry. In the past, scholars have paid more attention to the environmental biogeochemical behaviors and ecological and environmental effects of heavy metals, nutrients, and other pollutants in the soil/sediment and surface water in the coastal areas of the Yangtze estuary [31,32]. Furthermore, much research has focused on the groundwater in the Yangtze River Delta, but most studies examined the environmental biogeochemical behaviors of soil/sediment and heavy metals, nutrients and other pollutants, general groundwater quality, and seawater intrusion [15,33,34,35,36], indicating that groundwater in Qidong has been subject to severe overexploitation and seawater intrusion. Nonetheless, few studies have examined the chemical characteristics of shallow groundwater in the Yangtze River Delta and estuarine coastal areas, in which aquifers have been subject to the natural impacts of both the Yangtze River and the sea. In recent years, the extent of saltwater intrusion in the Yangtze River estuary has increased significantly due to the impoundment of the Three Gorges Dam [37]. Salinization is one of the important factors for reducing water quality. Knowledge on hydrochemical characteristics is crucial to assess the groundwater quality for understanding the suitability for domestic and irrigation purposes [38].

This paper examines the shallow groundwater in the Qidong City coastal area (an annular area 10 km from the coastline) as the research object, studying its geochemical characteristics and analyzing the influence of river and seawater on coastal areas in order to provide significant information to assist water resources management in coastal areas of Yangtze River estuaries, and other similar coastal areas globally. The purpose of this study is based on the chemical characteristics of shallow groundwater in a coastal plain environment to identify major differences in the water chemistries of such environments, to determine hydrochemical evolution mechanisms, and to assess the quality of shallow groundwater. The present work will be useful for making a database for the long-term use of groundwater, management, and the future strategy to protect the water resources of the region in changing climatic scenarios. It could enable planners and policy makers to develop strategies to mitigate the impact of external factors on shallow groundwater resources and improve the groundwater quality.

## 2. Materials and Methods

### 2.1. Study Area

**Figure 1:**
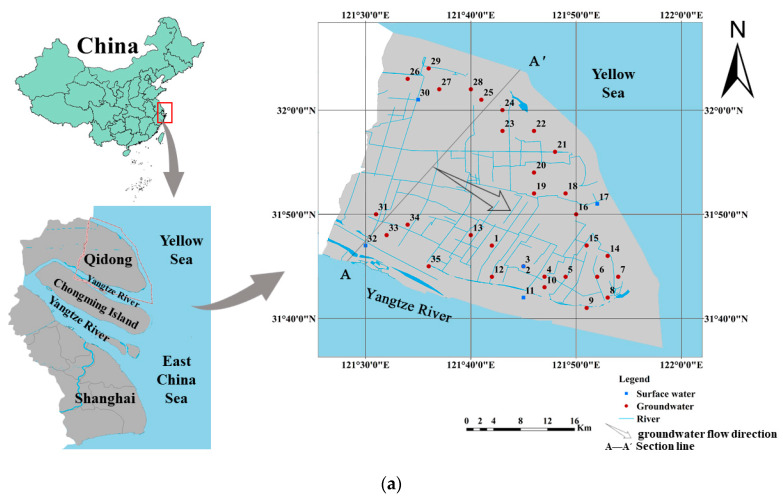

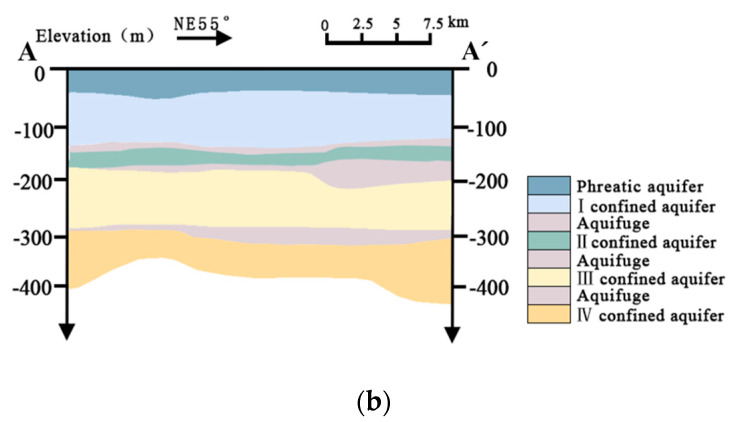
Location of study area and well sampled in the littoral zone of Qidong (**a**), hydrogeological cross section of study area (**b**) (modified from [39]).

The coastal area of Qidong City, north of the Yangtze River estuary, was selected as the study area (Figure 1). Qidong City lies in the easternmost part of Jiangsu Province, which is located in southeastern China, belonging to the Yangtze River Delta hydrogeological and sedimentary area. The southwestern part of the study area, situated in the north wing of the Yangtze River estuary, has a geomorphology dominated by a fluviatile coastal plain. The eastern and northern parts of this area are marine plain, which is on the verge of the Yellow Sea, and the southern region is on the northern branch of the Yangtze River estuary.

The study area has a northern subtropical humid climate, with many characteristics of a maritime monsoon climate. Over the period 1981–2010, the average temperature was 15.6 °C, the average temperature of the coldest month was −1.9 °C, and the average temperature of the hottest month was 33.2 °C. The annual average precipitation was 1112.0 mm, of which the monthly precipitation from May to September was 697.3 mm, accounting for 62.7% of the annual precipitation.

The study area belongs to the Yangtze River Delta plain, which is a low-lying area with a surface elevation of 2.0–6.0 m above sea level. The topography of the micro-area is slightly undulating, with a slight dip from northwest to southeast. The north–south gradient is about 1/30,000 m and the gradient of the east–west is about 1/43,500 m. Therefore, it is speculated from a general perspective that the groundwater in this region is transported from northwest to southeast and discharged into the sea in the coastal area.

The study area is covered by Quaternary strata and belongs to the hydrogeological zone of the Yangtze River Delta. With the influence of the Yangtze River and the Yellow Sea, it is mainly composed of coastal and estuarine sediments, including littoral-marine (mostly sands) and fluvial-deltaic material (mostly silt, clay, sand, and gravel). Furthermore, the aquifers in the study area are composed of Quaternary Holocene sediments with a thickness of 200–300 m and are characterized by a multi-cycle paralic-sedimentary deposit. This thick and loose strata with a multi-sand layer and good permeability provides favorable conditions for the formation of pore groundwater so that large amounts of loose rock pore water, with a complex water quality and multiple layers, are widely distributed in the littoral zone. According to the age of aquifer, the characteristics of aquifer medium, hydraulic properties, and the depth of groundwater circulation, loose pore water in the area can be divided into three aquifer systems. From top to bottom, it is the Holocene aquifer system (saturated aquifer), the Upper and Middle Pleistocene aquifer system (Ⅰ–Ⅲ confined aquifers), and the Early Pleistocene aquifer system (Ⅳ–Ⅴ confined aquifer), respectively [33]. Since mainly shallow groundwater samples were collected in this study, and the depth of the sample wells are 4.5–5 m, the groundwater samples collected are mainly from the Holocene aquifer system, which is a saturated aquifer. Therefore, this article will introduce the hydrogeological characteristics of the saturated aquifer.

The saturated aquifers of the study area are paralic sediments. The lithology of it could be divided into upper and lower sections: the upper section is interbedded by silty sand, sabulous clay and loam, the lower section is loam mixed with powdery sand [33,40]. The saturated aquifer floor has a buried depth of 15–35 m, with a thickness of about 15–35 m, and the buried depth of groundwater table is 1–3 m. The horizontal and vertical hydraulic conductivity of the saturated aquifer are 3 × 10^−4^–8 × 10^−4^ m/d and 3 × 10^−5^–8 × 10^−5^ m/d, respectively [41]. The water inflow in a single well is generally 100 m^3^/d [33].

In natural conditions, saturated water table is strikingly controlled by topography. The overall flow direction of the shallow groundwater in study area is from northwest to southeast, and the hydraulic gradient is 0.04‰–2‰. Due to the gentle terrain, the horizontal runoff of shallow groundwater is very slow, with an average runoff velocity of 0.2 mm/d [42]. Owing to the shallow buried depth of saturated water, this area is mainly recharged by atmospheric precipitation, river channel infiltration, agricultural irrigation return flow, lateral runoff (from the sea area), and overflow recharge. Most of the surface runoff in the study area is derived from the Yangtze River. Additionally the water table of the Yangtze River is affected by tides. As the tide rises, the shallow groundwater is recharged by the Yangtze River, while the shallow groundwater is discharged into the Yangtze River when the tide sets out [43]. Groundwater is mainly discharged by evaporation or subsurface current to the sea. Artificial discharge is also an important way for groundwater to discharge. Most geological surveys have confirmed that the strata in this area are rich in calcite, potassium feldspar, plagioclase but there are basically no evaporative minerals (such as gypsum and halite) [43].

### 2.2. Sampling and Measurement

On August 21–22, 2017, local domestic wells were used as sampling sites for shallow groundwater and the rivers water were as surface water samplings within 5–10 km from the shoreline of Qidong City. A total of 35 water samples were collected in the study area, including 30 groundwater samples and five surface water samples. Temperature (T) and pH were measured on site, and groundwater sampling was done once field parameters were stabilized. All the groundwater samples collected for chemical analysis were filtered in the field using an ADVANTEC (Collins, Shanghai, China) 0.2 µm cellulose-acetate filter before storing in sampling bottles.

All sampling bottles were rinsed with tap water before sampling and soaked in 10% nitric acid, then rinsed with tap water and deionized water. When taking a water sample, the bottle was rinsed 2–3 times with sampling water. The sample bottles were put into portable refrigerators for storage, and the samples were sent to Shanghai SEP Analytical Services Co., Ltd., for analysis. The method for collecting and storing groundwater samples was followed in accordance with the Water Sample Collection and Storage Method (HZ-DZ-DXS-0002). The groundwater samples were analyzed for total dissolved solids (TDS), total hardness (as CaCO_3_), major elements (Na^+^, K^+^, Ca^2+^, Mg^2+^, Cl^−^, SO_4_^2−^, HCO_3_^−^, NO_3_^−^, NH_4_^+^), and micro-components (Fe^2+^, Mn^2+^, Cu^2+^, Al^3+^, Zn^2+^, Hg^2+^, Pb^2+^, As^3+^, Cd^2+^, Cr^6+^, NO_2_^−^, and F^−^). The TDS and total hardness were determined by weighing method and EDTA titrimetric method, respectively. The contents of Na^+,^ K^+^, Ca^2+^, Mg^2+^, Fe^2+^, and Hg^2+^ were performed by plasma emission spectrometry. The contents of Mn^2+^, Cu^2+^, Al^3+^, Zn^2+^, Hg^2+^, Pb^2+^, As^3+^, Cd^2+^ were carried out using inductively coupled plasma mass spectrometry. The content of NH_4_^+^ and Cr^6+^ were detected by Nessler’ reagent spectrophotometry and 1, 5-diphenycarbohydrazide spectrophotometry, respectively. Anion concentrations were estimated using standard analytical methods. All water quality parameters were analyzed based on common and standard methods intended for the examination of a variety of water qualities.

In addition, ionic balances (IB error in Table 1) were undertaken on the chemical analyses for quality control purposes [44]. The major ions (Na^+^, K^+^, Ca^2+^, Mg^2+^, Cl^−^, SO_4_^2−^, HCO_3_^−^, NO_3_^−^) of groundwater samples were used to compute the IB error. Overall error values below ±5% are the best analytical estimation and the accepted error level is no more than ±10% [25]. The IB error values of samples varies between −9.71% and 8.60%, with an average value of 0.36% (Table 1). A total of 80% of groundwater samples in study area had IB error values less than ±5%. The balance errors were accurate.

In the statistical calculations and graphical representations, the non-detected values were substituted with the half of detection limit (DL) value. All statistical analyses were performed with the SPSS 13.0 software. The spatial distributions for groundwater major parameters containing TDS, Na^+^, Cl^−^, Ca^2+^, HCO_3_^−^, Mg^2+^, SO_4_^2−^, NO_3_^−^, and NH_4_^+^ were completed with the support of spatial analyst modules in ArcGIS 9.2 software.

## 3. Results

### 3.1. Hydrochemical Constituents of Shallow Groundwater

The statistical analysis of the hydrochemical elements and microelements of shallow groundwater is shown in Table 1.

As shown in Table 1, the temperature of shallow groundwater variation ranged from 22.0 to 25.6 °C with an average value of 24.03 °C. The TDS values ranged from 305 to 2520 mg/L with a mean value of 981 mg/L. The samples sites #22 and #29 had the highest TDS values at 2520 and 2510 mg/L, respectively. Additionally, the pH of the shallow groundwater in the study area varied only slightly, ranging from 7.79 to 8.19, indicating the groundwater is slightly alkaline. The total hardness (TH) of the shallow groundwater (calculated as CaCO_3_) in the study area ranged from 152.2 to 708.8 mg/L, with an average of 383.9 mg/L. The total hardness at three locations was extremely high, greater than 550 mg/L. They were sites #26, #27, and #29, and the contents were 575, 560, and 709 mg/L, respectively.

As for the major ions in groundwater, the average values of the Na^+^, K^+^, Ca^2+^, Mg^2+^, and NH_4_^+^ in groundwater were 212.18, 28.09, 66.13, 46.43, and 0.36 mg/L, respectively. Additionally, the HCO_3_^−^, Cl^−^, SO_4_^2−^, and NO_3_^−^ had the average values that 557.13, 209.23, 83.93, 7.32 mg/L, respectively. Table 1 shows the average concentration of cations and anions, arranged from highest to lowest, respectively: Na^+^ > Ca^2+^ > Mg^2+^ > K^+^ > NH_4_^+^ and HCO_3_^−^ > Cl^−^ > SO_4_^2−^ > NO_3_^−^. As for the microelements in groundwater, the contents of Cu^2+^, Al^3+^, Zn^2+^, Hg^2+^, Pb^2+^, As^3+^, Cd^2+^, and Cr^6+^ in groundwater samples were very low, especially the Cr^6+^ of all samples was not detected. The value of NO_2_^−^ in the groundwater ranged from 0.0025 mg/L to 1.49 mg/L with an average value of 0.158 mg/L. The concentration of Fe^2+^ was ranged from 0.025 to 3.11 mg/L with a mean value of 0.29 mg/L. The average concentration of Mn^2+^ was 0.108 mg/L and maximum value of 0.654 mg/L. The content of F^-^ was observed from 0.21 and 0.91 mg/L with an average value of 0.414 mg/L.

The coefficient of variation (CV), a dimensionless quantity, is used to characterize the variation of spatial scale in hydrogeological variables and provide a measure of the variability of a sample without reference to the scale of the data. It can provide clues about the determinants of the geographical distribution of major elements. A coefficient of variation ranging from 0% to 10% generally indicates a weak variation, 10% < CV ≤ 100% means a medium variation, and CV > 100% suggests a strong variation. When hydrochemical elements have a CV greater than 100%, they have likely been affected by external environmental conditions, such as hydrogeological conditions, topography, hydrometeorology, and human activities, reflecting large spatial changes in groundwater content and indicating significant sensitivity to environmental changes [45].

By analyzing the variability of each major ion, it can be concluded that the CV of Ca^2+^, Mg^2+^, K^+^, SO_4_^2−^, and HCO_3_^−^ was relatively small and stable, indicating that these five ions are less affected by human activities, and more by the natural environment. Furthermore, Na^+^, Cl^−^, NO_3_^−^, and NH_4_^+^ had a strong overall spatial variability with CVs greater than 100%, especially NH_4_^+^, which had a CV value of 208.3% indicating that these four ions are greatly affected by human activities and the natural environment. The CV values of Na^+^ and Cl^-^ were 117.2% and 143.8%, respectively, indicating a strong variability. It can be inferred from the geographical location of the research area that the distribution of sampling points is concentrated on the Yangtze River bank or on the eastern seashore bank. Therefore, the groundwater could be under the influence of seawater mixing. The high CV of NO_3_^-^ and NH_4_^+^ indicates that some sampling sites are significantly affected by pesticides, fertilizers, and livestock waste, causing large spatial variations [46].

Correlation analysis can reveal the similarity and heterogeneity of groundwater hydrochemical parameters and reflect similarities and differences in their origins. A Spearman correlation and regression analysis were performed with SPSS 13.0 software. Statistically significant effects were defined as *p* < 0.01. It can be seen from Table 2 that TDS is prominently correlated with Na^+^, K^+^, Mg^2+^, SO_4_^2−^, HCO_3_^−^, and Cl^−^, indicating the significant contributions of these elements to shallow groundwater mineralization. There was a significant correlation between Cl^−^ and the major ions (K^+^, Na^+^, Mg^2+^, and HCO_3_^−^), with the strongest correlation found between Cl^−^ and Na^+^ (0.936). The strong correlation between Cl^−^ and Na^+^ implies that they share the same origin. The chemical composition of shallow groundwater in this area is significantly affected by seawater intrusion. The good correlation between K^+^ - Na^+^ and Ca^2+^ - Mg^2+^ manifested that they were influenced by the same factors. HCO_3_^−^ is poorly correlated to Ca^2+^ indicating another source other than the calcite dissolution. The poor correlation between Na^+^ and Ca^2+^ is mainly due to the fact that the area is located at the coastal sea and land junction, and has been affected by seawater and land freshwater for a long time. It is easy to be affected by the external environment and cause water–rock ion exchange in the aquifer [33]. Additionally, there was a low correlation between Ca^2+^ and SO_4_^2−^, which revealed the concentrations of these two ions were controlled by mechanisms other than the dissolution of the gypsum. It confirmed the absence of evaporative minerals in the study area. NO_3_^−^ was negatively correlated with NH_4_^+^ and NO_2_^−^ while NH_4_^+^ was positively correlated with NO_2_^−^, reflecting the complicated transformation of three nitrogens. NO_3_^−^ was negatively correlated with Fe^2+^ and As^3+^. The positive correlation existed in Fe - NH_4_^+^, Fe - NO_2_^−^, Mn - NH_4_^+^, Cu - NO_3_^−^, Zn - Al, and Fe - As.

### 3.2. Hydrochemical Types of Shallow Groundwater and Its Spatial Distribution

**Figure 2:**
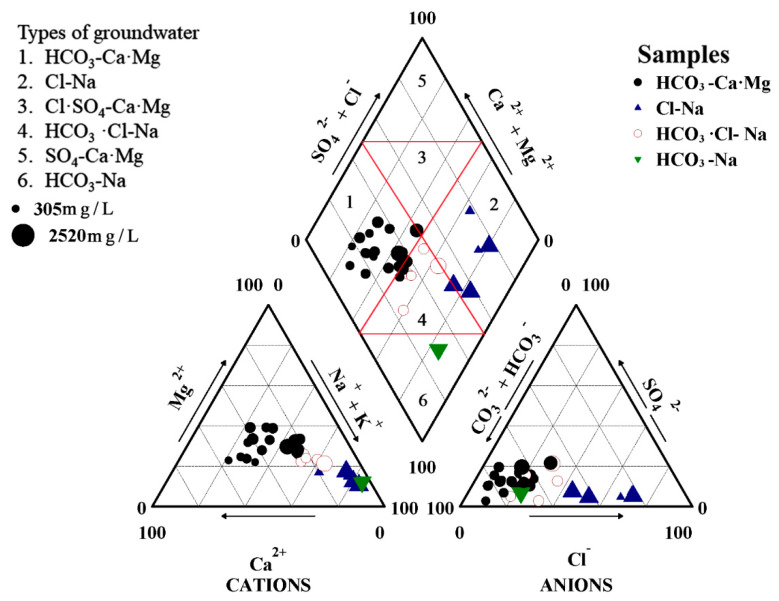
Piper diagram of shallow groundwater. The size of symbols represents the TDS (total dissolved solids).

Based on the hydrochemical data from shallow groundwater in Qidong City, the major ion (Na^+^, K^+^, Ca^2+^, Mg^2+^, HCO_3_^−^, CO_3_^2−^, SO_4_^2−^, Cl^−^) concentrations in shallow groundwater samples were plotted in a trilinear Piper diagram [47] to determine the geochemical characteristics of groundwater in the study area. The Piper diagram (Figure 2) shows that Na^+^ is the main cation while HCO_3_^−^ dominates in anions. Furthermore, there is a wide range of Cl^−^ and less SO_4_^2−^, and a clear trend in cations. The groundwater samples in the study area were classified into four major hydrogeochemical facies, which are HCO_3_-CaMg (black circle), Cl-Na (blue triangle), HCO_3_Cl-Na (red hollow circle), and HCO_3_-Na (green inverted triangle) types. The majority of the groundwater samples, represented by black circles, occupying the left part of the central diamond graph were determined to be HCO_3_-CaMg water, and 63% of the total groundwater samples were assigned to this category. The Cl-Na water type scattered in the right half of the diamond plot constituted about 17% of the samples (#7, #8, #14, #22, and #29), marked by blue triangles. Samples #2, #12, #19, #21, and #24, which are shown as hollow circles in the central diamond plot, belong to a HCO_3_·Cl-Na water category; the groundwater composition of sample #25, shown as green inverted triangle, is HCO_3_-Na. To sum up, the prevalent type of water in the study area is HCO_3_-CaMg, followed by Cl-Na, and HCO_3_·Cl-Na. Compared to the other samples, these Cl-Na water type samples were found closer to the sea coast, where groundwater may have been impacted by seawater intrusion in the shallow aquifer. The groundwater consists of HCO_3_-CaMg and HCO_3_-Na, evolving closer to the coastal strip into HCO_3_·Cl-Na and Cl-Na, due to the transition of major anions from bicarbonate to bicarbonate chloride and chloride along groundwater pathways, which demonstrates that the quality of shallow groundwater along the coast is affected by seawater intrusion.

**Figure A1:**
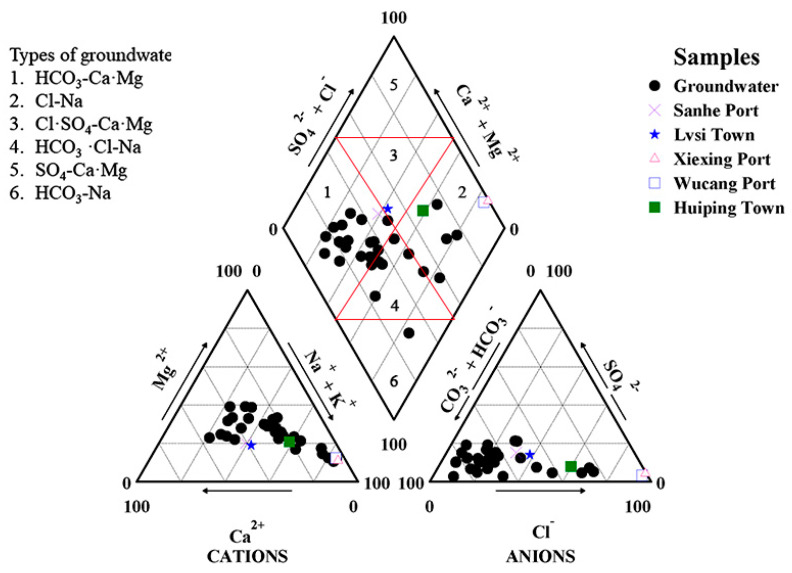
Piper diagram of groundwater and surface water.

The comparison of chemical characteristics between surface water and shallow groundwater is significant to understand the impact of surface water on groundwater. It can be seen from Table A1 in Appendix A that the concentrations of major hydrochemical parameters, except NO_3_^−^ and TDS in Wucang Port (#11) and Xiexing Port (#17), were significantly higher than those the three other surface water samples, Huiping Town (#3), Lvsi Town (#30), and Sanhe Port (#32). In addition, the water chemistry Piper diagram of surface water and groundwater (Figure A1) intuitively reveals that significant spatial difference in water chemistry characteristics of surface water. The Wucang Port (#11) and Xiexing Port (#17) anions were completely dominated by Cl^−^. The water chemistry type is chloride-sodium (Cl-Na). Wucang Port (#11) is located in the lower reaches of the Yangtze River, which influenced by seawater mixing processes, while Xiexing Port (#17) is located on the East China Sea coast, so it already contains saltwater. The chemical characteristics of these surface water samples are roughly identical with those of shallow groundwater surrounding it, confirming the impact of seawater intrusion on water quality in the region. On the other hand, the other three surface water have different water types, HCO_3_-CaMg. The differences could be explained by the fact that Sanhe Port (#32) is situated in the lower reaches of the Yangtze River, upstream of Wucang Port (#11), and it is less affected by the seawater mixing than Wucang Port (#11). Furthermore, the surface water samples of Lvsi Town (#30) and Sanhe Town (#32) are located inland, and all concentrations were approximately consistent with that of shallow groundwater which is adjacent to the surface water samples, confirming the influence of the phenomena of freshening to the shallow groundwater under natural recharge.

### 3.3. Geospatial Distribution of Key Ions in Coastal Zone

**Figure 3:**
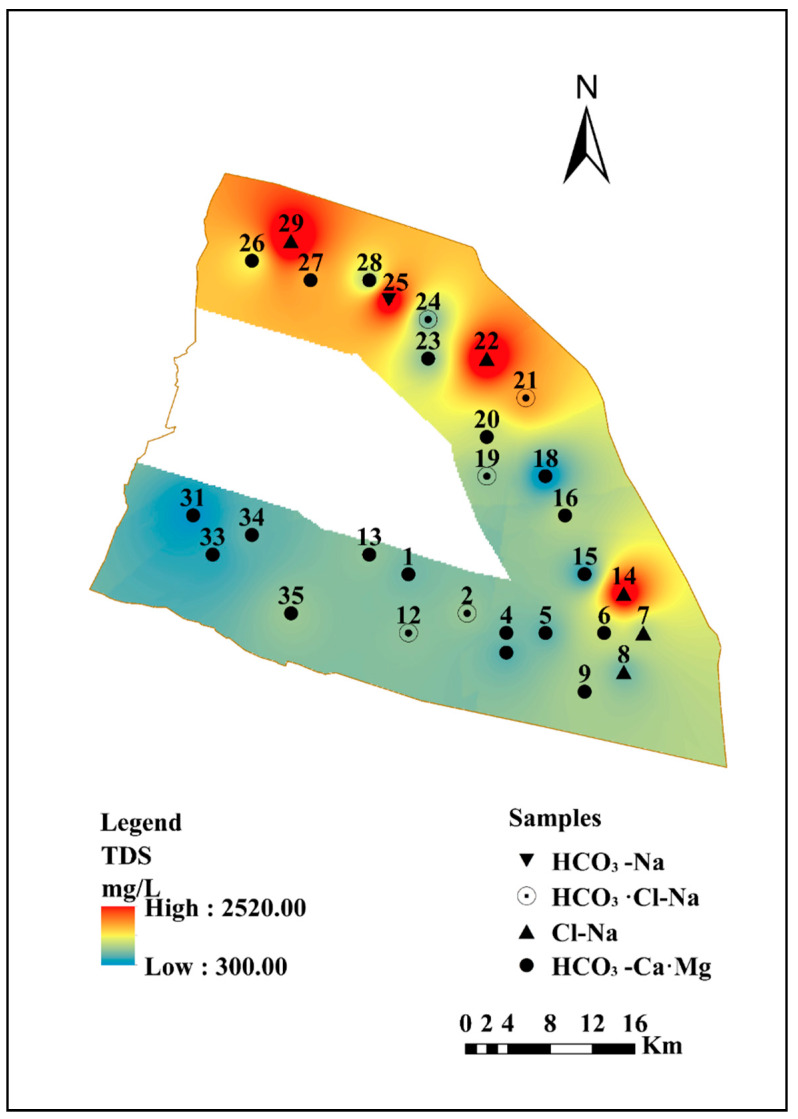
Geospatial distribution of TDS in the study area.

**Figure 4:**
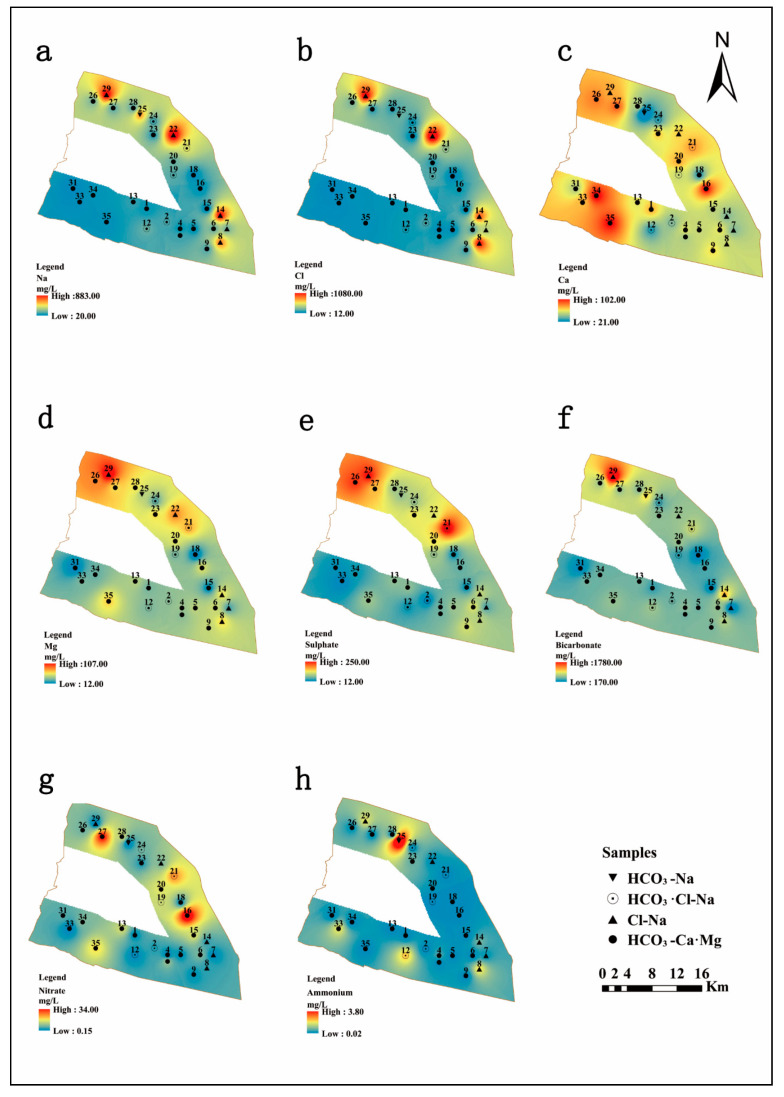
Spatial distribution of the major ion concentrations (mg/L) in study area: (**a**) Na, (**b**) Cl, (**c**) Ca, (**d**) Mg, (**e**) SO_4_^2−^, (**f**) HCO_3_^−^, (**g**) NO_3_^−^, (**h**) NH_4_^+^.

The geological interpretation of key factors may help to determine the spatial distribution of hydrochemical variables in groundwater, which is governed by complicated impact processes. In this study, the major hydrochemical parameters (TDS, Na^+^, Cl^−^, Ca^2+^, HCO_3_^−^, Mg^2+^, SO_4_^2−^, NO_3_^−^, NH_4_^+^) were gridded using a GIS-based inverse distance weighted (IDW) technical approach to demonstrate the corresponding spatial distribution models (Figure 3 and Figure 4), which are shown as maps.

TDS generally refers to the contents of various components present in the water, including compounds, molecules, and ions [48]. The large variation in TDS concentrations is influenced by diverse processes such as ocean water spray, ions from intruding seawater, seawater and estuarine alluvial aquifers, the influx of river water recharging the surrounding aquifer, mixed ion exchange processes along the groundwater flow paths, as well as geological influences, mineralogy diversification, and the geochemical environments. According to the data, most of the shallow groundwater samples on the shore of Qidong City were freshwater (TDS < 1000 mg/L), and only a small portion (approximately 23%) of the total sampling area was brackish water (1000 < TDS < 3000 mg/L), which are presented as #14, #21, #22, #26, #25, #27, and #29. The TDS value gradually increased from southwest to northeast, as shown in Figure 2. In combination with the geographical position of the research area, the south bank of the coastal shore of Qidong City is located in the lower reaches of the Yangtze River, and along the Yangtze River, the further east towards the sea, the greater the contribution of seawater mixing in the shallow groundwater, resulting in an increase in TDS content. TDS, Na^+^, and Cl^−^ (Figure 3 and Figure 4a,b) had similar distribution patterns, where they were high in the northern, northeastern, and southeastern coastal regions, which suggests that Na^+^ and Cl^−^ may be derived from the same source of intruded modern seawater, which has a significant impact on groundwater salinity. The highest values for TDS, Na^+^, and Cl^−^ were exhibited at site #22. Mg^2+^ concentrations in the northwestern of the area were much higher than those in the other regions. Ca^2+^ displayed a different trend, with the concentrations being high in the west of the study area. However, sample site #16, located in the eastern margin, had the highest value. It can be seen in Figure 4c,d that the distribution of Ca^2+^ and Mg^2+^ is irregular. The concentration of SO_4_^2−^ increased gradually from south to north of the study area, and the three highest values of SO_4_^2−^ occurred at sample sites #21, #26, and #29. In addition, there was a similar distribution between the Mg^2+^ and SO_4_^2−^, with the high concentrations found in the north of the region along the Yellow Sea coast. Due to the high concentration of Mg^2+^ and SO_4_^2−^ in seawater, and the fact that there were no gypsum minerals present in the study area based on previous geological data, the high content of Mg^2+^ and SO_4_^2−^ in the coastal area was most likely a result of seawater intrusion. The SO_4_^2−^ may also be affected by natural processes and artificial pollution, like the breakdown of organic substances in weathered soils, sulfate fertilizers, and domestic wastewater [49].

It was noted that the HCO_3_^-^ ion distribution map (Figure 4f) showed a distinguished enrichment towards the north and the highest value, 1050 mg/L, was recorded at #14. Nitrate and ammonia concentrations were generally low (most NO_3_^−^ < 10 mg/L, most NH_4_^+^ < 0.5 mg/L). The sample sites #16 and #27 were the two locations with the highest NO_3_^−^ concentrations, and the highest NH_4_^+^ value was 3.73 mg/L at sample site #25. The sample sites #16, #25, and #27 are located in the concentrated residential areas, so that the extraordinarily high nitrate and ammonia concentrations are associated with anthropogenic pollution [50].

## 4. Discussion

### 4.1. Hydrochemical Evolution Mechanisms

**Figure 5:**
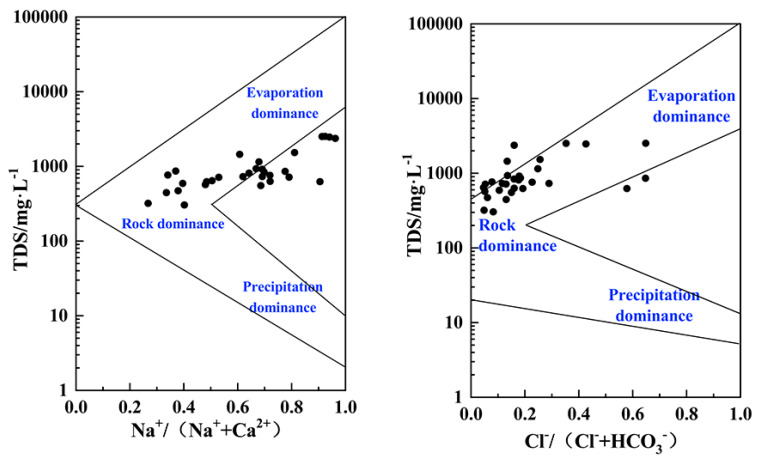
Gibbs diagram of shallow groundwater.

The Gibbs diagram could provide insightful guidance on the formation mechanisms of the hydrochemistry of groundwater. There are three main mechanisms for controlling the hydrochemical composition of natural water, which are atmospheric precipitation, rock weathering hydrolysis, and evaporation concentration, identified from the Gibbs diagram [20]. As shown in Figure 5, the shallow groundwater in this area has a TDS range of 100–5000 mg/L, the Na^+^/(Na^+^ + Ca^2+^) ratio is 0.2–1.0 overall, and the ratio of Cl^−^/(Cl^−^ + HCO_3_^−^) is generally between 0 and 0.7. The points were mainly distributed in the upper middle part of the Gibbs diagram. It can, therefore, be concluded that the compositions of shallow groundwater in the study area are predominantly controlled by a combination of evaporation and weathering dissolution of minerals. Moreover, some of the points did not fall completely in line with the Gibbs’ classification, indicating that, in addition to the three impact mechanisms mentioned above, there are other factors influencing groundwater chemical composition in the coastal study area. Due to the unique geographical location of the study area, the shallow groundwater in the zone was not only recharged by the freshwater of the Yangtze River but was also impacted by seawater from the East China Sea. It cannot be ignored that the mixing of salty and freshwater is one significant formation mechanism. Complex hydrogeochemical processes in the shallow aquifer ultimately determine the current composition of groundwater.

**Figure 6:**
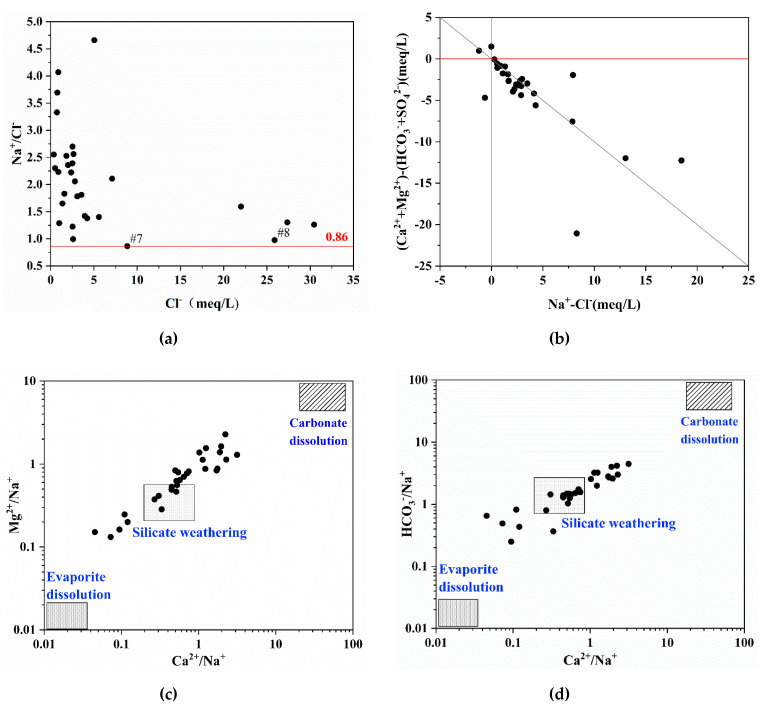
Scatter diagram of (**a**) Na^+^/Cl^−^ vs. Cl^−^, (**b**) [(Ca^2+^ + Mg^2+^) − (HCO_3_^−^ + SO_4_^2^^−^)] vs. [Na^+^-Cl^−^], (**c**) Mg^2+^/Na^+^ vs. Ca^2+^/Na^+^, and (**d**) HCO_3_^−^/Na^+^ vs. Ca^2+^/Na^+^.

The ions’ proportionality coefficient is used for the analysis of groundwater evolution and hydrogeochemical processes. The value of Na^+^/Cl^−^ (meq/L) is known as the groundwater genetic coefficient and is widely used to characterize the degree of Na^+^ enrichment in groundwater and for the research on saline intrusion mechanisms in coastal regions [3]. The standard value of Na^+^/Cl^−^ (meq/L) in seawater is 0.86 [51]. Chloride is a conservative element, on which chemical reactions in groundwater and seawater have minimal influence. There are three conclusions that may be drawn from the distribution of the scatter points. Firstly, when the ratio of Na^+^/Cl^−^ was near 0.86 it indicated that the salinity in shallow groundwater was most likely derived from the mixing of seawater. Secondly, when the ratio of Na^+^/Cl^−^ < 0.86, it indicated that the shallow groundwater is in a concentrated metamorphic condition and has the characteristics of paleo-sedimentary water. Thirdly, when the ratio of Na^+^/Cl^−^ > 0.86, the milliequivalent concentration of Na^+^ was greater than Cl^−^, and the shallow groundwater was likely impacted by the weathering dissolution of silicate minerals. From the data analysis, it can be deduced that Na^+^/Cl^−^ in the study area ranged from 0.87 to 4.67, and the average value was 2.09, which is greater than the value for standard seawater of 0.86. Samples of #7, #8, and #16, with Na^+^/Cl^−^ = 0.86 and located in the southeastern of coastal area, are strongly affected by the modern seawater intrusion. The scatter diagram of the Na^+^/Cl^−^ ration versus Cl^−^ (refer to Figure 6a) indicates that most of the groundwater samples can be plotted above the line of Na^+^/Cl^−^ = 0.86, which could suggest that the shallow groundwater in the study area was not only affected by a combination of seawater mixing but also probably by the weathering and dissolution of silicate minerals.

In order to further determine the influence of weathering dissolution of minerals on the hydrochemical evolution in the study area, the Ca^2+^/Na^+^, Mg^2+^/Na^+^ and HCO_3_^−^/Na^+^ were used to identify the carbonate, silicate, and evaporite minerals dissolution [52]. In the scatter diagram of the relationship Ca^2+^/Na^+^ relative to Mg^2+^/Na^+^ and Ca^2+^/Na^+^ relative to HCO_3_^−^/Na^+^ (Figure 6c,d), these major cations ratios (meq/L) of groundwater were mainly distributed between the weathering of silicate and dissolution of carbonate minerals. It indicates that the weathering dissolution of silicate and carbonate accounts for the contribution to the hydrochemical evolution of the study area. This finding is in good agreement with the inferences drawn from the Gibbs diagram (Figure 5).

The cation exchange process is another vastly essential process in coastal aquifers, which contributes to the evolution of groundwater composition. Additionally, the negative correlation of Na^+^ and Ca^2+^ implies the cation exchange process prevailing in the study aquifer. On the other hand, the hydrogeological basin of the Yangtze River Delta alluvial plain is regarded as a favorable environment for the cationic exchange processes in the clay matrix of this aquifer because of the occurrence of montmorillonite clay, which has the good ability to adsorb cations. The scatter diagram (Figure 6b) of the relationship [(Ca^2+^ + Mg^2+^) − (HCO_3_^−^ + SO_4_^2−^)] relative to (Na^+^-Cl^−^) could determine whether the exchange of Na^+^ against (Ca^2+^ or Mg^2+^) is occurring through the clay matrix [5,53]. In general, if there is absence of cation exchange, all groundwater samples will be placed in the origin of the diagram. Otherwise, the cationic exchange process controls the chemical parameters of the water, and the relationship between these parameters should be linear with a slope equal to −1 [19,54]. In consideration of salinity signature, the mixing mechanism is mainly caused by seawater intrusion and the freshening phenomena is due to the cation exchange reaction in the aquifer matrix, in which the clay mineral is the cationic exchanger [55].

The following Equations (1) and (2) show the gain or loss related to Na^+^ and (Ca^2+^ + Mg^2+^) within the exchanger X.

Na-X + 1/2Ca^2+^→Na^+^ + 1/2Ca-X, (1)

represents the freshening.

1/2Ca-X + Na^+^→1/2Ca^2+^ + Na-X, (2)

represents the seawater intrusion.

The scale diagram of [(Ca^2+^ + Mg^2+^) - (HCO_3_^−^ + SO_4_^2-^)] vs. (Na^+^-Cl^−^) in Figure 6b shows that most of the groundwater samples scatter around the line of y = −x. These shallow groundwater samples are the results of freshening phenomenon under natural recharge conditions and the recharge water tends to flush out the brackish water along the flow path toward the discharge zone [56]. There are samples in Figure 6b concentrated in the origin, which indicated no cation exchange occurs. The reason is the evaporation process followed by carbonate precipitation [57]. Additionally, the cation-exchange process could be characterized by chloro-alkaline index [58]. The calculation formula of chloro-alkaline index CAI-1, CAI-2 is as follows (all ionic concentrations are expressed by meq/L):

CAI-1 = [Cl^−^-(Na^+^ + K^+^)]/Cl^−^, (3)

CAI-2 = [Cl^−^-(Na^+^ + K^+^)]/ (HCO_3_^−^ + SO_4_^2−^ + NO_3_^−^). (4)

**Figure 7:**
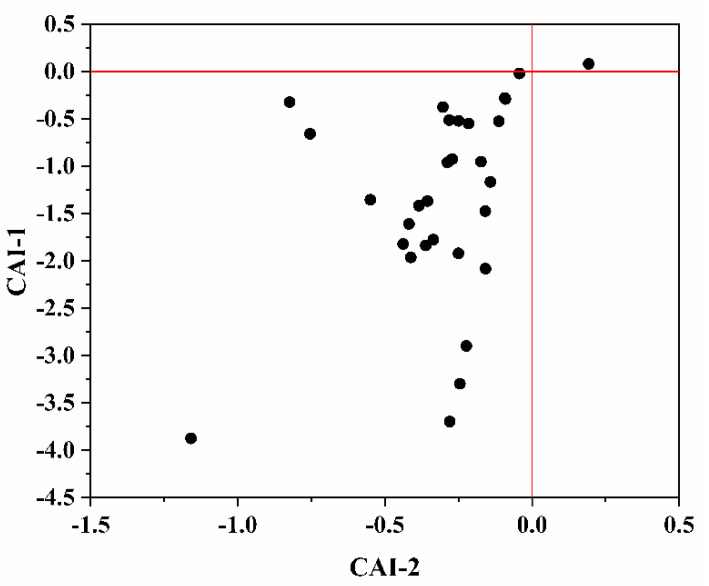
Chloro-alkaline indices of shallow groundwater.

If both CAI-1 and CAI-2 are negative, this indicates that Ca^2+^ or Mg^2+^ in the groundwater is exchanged with Na^+^ in aquifer medium (Equation (1)), while if both CAI-1 and CAI-2 are positive, this implies that reverse cation exchange occurs (Equation (2)). As shown in Figure 7, most chlor-alkali indexes are less than 0, exhibiting that exchange between Ca^2+^ and Mg^2+^ in groundwater and Na^+^ in water-bearing media mainly prevails in the study area. The same result is also noted using the relationship [(Ca^2+^ + Mg^2+^) − (HCO_3_^−^ + SO_4_^2−^)] relative to (Na^+^-Cl^−^) (Figure 6) and Gibbs diagrams (Figure 5).

**Figure 8:**
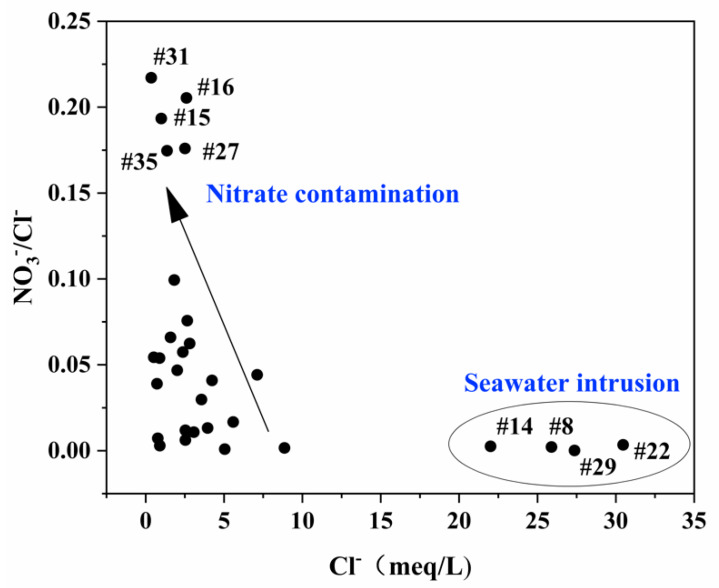
Scatter diagram of NO_3_^−^/Cl^−^ vs. Cl^−^.

As the saturated aquifer of Yangtze River estuarine coastal area supports irrigation activity and the relatively concentrated residential area in coastal areas, the study area is subject to contamination by nitrate which cannot be ignored. The plot of ratio NO_3_^−^/Cl^−^ versus Cl^−^ (Figure 8) could be used as an indicator to identify seawater intrusion and anthropogenic pollution [59]. Saline water with high concentrations of Cl^−^ exhibited low values of NO_3_^−^/Cl^−^, confirming seawater intrusion. On the other hand, the increase of NO_3_^−^/Cl^−^ values with a decrease of Cl^−^ indicates the impact of anthropogenic activity on the shallow groundwater. The anthropogenic pollution may have a strong influence on the occurrence of NO_3_^−^ in the groundwater. Figure 8 shows that the shallow groundwater of sample sites #8, #14, #22, and #29 were influenced by seawater intrusion, and the sample sites #15, #16, #27, #31, and #35 were affected by nitrate contamination. Additionally, the inference is consistent with the geospatial distribution of nitrate (Figure 4) as mentioned above.

**Figure 9:**
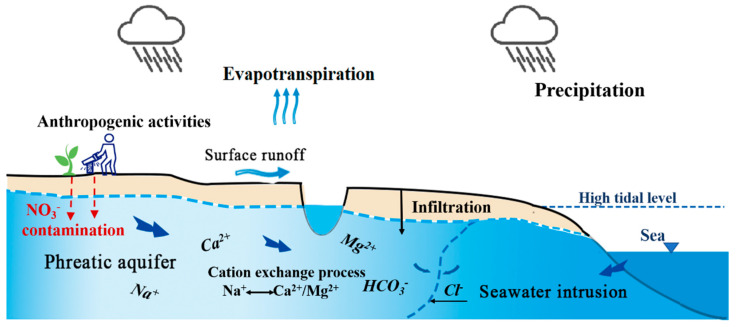
Conceptual model of the main hydrochemical evolution mechanisms recognized in the study area.

As the analysis above, the coastal aquifers are subjected to the ongoing influence of the seawater intrusion, ion exchange processes, freshwater infiltration, and mineral (carbonate and silicate) dissolution, which impact groundwater evolution. Additionally, the anthropogenic activities about agricultural pollution cannot be ignored. A summary of the main hydrochemical evolution mechanisms in the aquifer is depicted in Figure 9.

### 4.2. Evaluation of Groundwater Suitability in Drinking and Irrigation

According the World Health Organization (WHO) [60] standards, the shallow groundwater analytical results of pH, total hardness (TH), TDS, major ions, and microelements were classified for drinking purposes. As Table 3 shows, there are 20%, 40%, 13.3%, and 3.3% of Na^+^, K^+^, Cl^−^, and NH_4_^+^ concentrations in the entire shallow groundwater samples that exceed the WHO maximum allowable limit and the resulting undesirable effects on humans. For the microelements in shallow groundwater, it has been found that 20% of Fe, 30% of Mn, and 6.7% of As concentrations in all samples exceed the maximum allowable limit. The higher concentrations of Fe^2+^ were observed in the shallow groundwater samples #4, #12, #25, #26, #29, and #33. The content of Mn^2+^ in #4, #5, #7, #14, #23, #26, #27, #29, and #33 samples exceeded the limit of WHO standards. Additionally, the content of As^3+^ also in samples #29 and #33 exceeded the maximum allowable limit standard. According to analysis results, the Ca^2+^, Mg^2+^, SO_4_^2−^, NO_3_^−^, NO_2_^−^, Cu^2+^, Al^3+^, Zn^2+^, Hg^2+^, Pb^2+^, Cd^2+^, Cr^6+^, and F^−^ concentrations in shallow groundwater of the study area were within the maximum allowable limit in the entire samples.

To ascertain the suitability of groundwater for any purposes, it is vital to classify the groundwater depending on its hydrochemical properties based on its TDS values [44]. According to the WHO standards, 16.7% of the samples exceed the permissible limits, which is shown in Table 3. From Table 4, 76.66% of the total samples could be used for drinking and 23.33% could be used for irrigation [61]. The classification of TH reveals that 70% of groundwater samples belong to the category named very hard and 30% in the hard category, indicating that the groundwater in the study area is useless for laundry work [62]. The hard groundwater could cause scaling the contact surface, plug pipes, and irrigation lines and even cause foliar scale deposits [25]. The 23.3% of total groundwater samples based on the values of TH exceeds the maximum allowable limit of total hardness for drinking purposes as the reference of WHO standards. Referring to the WHO standards and classifications of TDS and TH, most of the groundwater aquifer seems to be unsuitable for safe drinking especially near the samples #4, #8, #14, #22, #25, #29, and #33. Water treatments and adequate processing are necessarily needed in advance if the groundwater is used for domestic potable water.

Excessive amounts of dissolved ions such as Na^+^, HCO_3_^−^, and CO_3_^2−^ in irrigation water adversely affect plants and soil texture, reducing the productivity of agriculture [63]. Salinity and indexes such as sodium absorption ratio (SAR) and soluble-sodium percentage (SSP) are important parameters for determining the suitability of groundwater for agricultural uses [10]. The relative activity of sodium ions in the exchange reaction with soil is expressed in terms of a ratio known as sodium adsorption ratio (SAR). SAR is an important parameter for determining the suitability of groundwater for irrigation because it is a measure of alkali/sodium hazard to crops [64]. Soluble-sodium percentage (SSP) is another important parameter which could assess whether groundwater contains excessive sodium, affecting the soil properties and reducing soil permeability.

SAR [65] is defined as follows (all ionic concentrations are expressed by meq/L):

SAR = Na^+^/[(Ca^2+^ + Mg^2+^)/2]^1/2^. (5)

Soluble-sodium percentage (SSP) [66] is obtained by the following (all ionic concentrations are expressed by meq/L):

SSP = Na^+^ × 100/(Na^+^ + Ca^2+^ + Mg^2+^ + K^+^). (6)

The classifications of groundwater samples with respect to SAR and SSP are shown in Table 4. The SAR values range from 0.64 to 18.53 with a mean value of 4.73. Most of the water samples, except #14, do not exceed the SAR value of 18, implying that the groundwaters within the study area are suitable for irrigation purposes. For the classification of SSP, out of 30 groundwater samples, 25 (83.33%) belong to the good to permissible; 14 (46.67%), excellent to good; three (10%), doubtful to unsuitable; two (6.67%), unsuitable categories. The SSP manifests that 93.3% of the samples are suitable for irrigation.

## 5. Conclusions

This paper focused on shallow groundwater of the estuarine coastal zone in Qidong, located in the Yangtze River estuary. The groundwater in coastal area is weakly alkaline. Na^+^ and Cl^−^ are the main factors affecting shallow groundwater salinization in the region. The prevalent water types are HCO_3_-CaMg, followed by Cl-Na and HCO_3_Cl-Na. The groundwater chemistry can be categorized as HCO_3_-CaMg and HCO_3_-Na, becoming HCO_3_Cl-Na and Cl-Na along the coastal strip. This finding demonstrates the impact of seawater mixing with the regional aquifer, as it undergoes a gradual freshening process.

The hydrochemical formation mechanisms of shallow groundwater were determined by combined effects of seawater intrusion and rock–water interaction (ion exchange processes, freshwater infiltration, and mineral dissolution). The intrusion of seawater in the coastal aquifer is the principal processes, but the freshening phenomenon in the discharge zone and the influence of anthropogenic activities are important and cannot be ignored.

The groundwater in study area is unsuitable for drinking based on the WHO standards and classifications of TDS and TH, as well as useless in domestic purposes unless water treatments have been undertaken in advance. On the other hand, the SAR and SSP demonstrate that more than 90% of the groundwater samples are suitable for irrigation.

## Figures and Tables

**Table 1:** Statistical summaries of hydrochemical elements of shallow groundwater in the study area.

Indicators	Mean Value	Standard Deviation	Minimum	Maximum	Variance (%)
T (℃)	24.03	0.96	22.0	25.6	4.00
pH	8.00	0.12	7.79	8.19	1.50
TH (mg/L)	383.91	127.98	152.15	708.71	33.30
TDS (mg/L)	981.03	650.62	305.00	2520.00	66.30
Na^+^ (mg/L)	212.18	248.68	20.90	883.00	117.20
K^+^ (mg/L)	28.09	20.08	5.39	76.30	71.50
Ca^2+^ (mg/L)	66.13	18.85	21.30	102.00	28.50
Mg^2+^ (mg/L)	46.43	20.33	12.20	107.00	43.80
Cl^−^ (mg/L)	209.23	300.95	12.62	1080.00	143.80
HCO_3_^−^ (mg/L)	557.13	297.53	170.85	1780.00	53.40
SO_4_^2−^ (mg/L)	83.93	58.12	12.54	250.00	69.20
NO_3_^−^ (mg/L)	7.32	7.96	0.16	33.08	108.70
NH_4_^+^ (mg/L)	0.36	0.75	0.01	3.73	208.30
NO_2_^−^ (mg/L)	0.158	0.296	0.0025	1.490	187.79
Fe^2+^ (mg/L)	0.290	0.60	0.025	3.110	207.06
Mn^2+^ (mg/L)	0.108	0.14	0.002	0.654	131.97
Cu^2+^ (mg/L)	0.0020	0.0028	0.00005	0.0138	142.45
Al^3+^ (mg/L)	0.214	0.198	0.0003	0.061	92.87
Zn^2+^ (mg/L)	0.012	0.011	0.003	0.051	95.57
Hg^2+^ (mg/L)	0.00011	0.00006	0.00005	0.00023	54.54
Pb^2+^ (mg/L)	0.00032	0.00024	0.00003	0.00129	74.77
As^3+^ (mg/L)	0.0033	0.0074	0.00004	0.0354	223.119
Cd^2+^ (mg/L)	0.00013	0.000031	0.000066	0.00018	24.75
Cr^6+^ (mg/L)	ND	ND	ND	ND	-
F^−^ (mg/L)	0.414	0.141	0.21	0.91	33.98
IB error (%)	0.36	4.20	−9.71	8.60	-

IB—ion balance; ND—non-detected.

**Table 2:** Correlation matrices of hydrochemical parameters of shallow groundwater.

	**pH**	**TH**	TDS	Na^+^	K^+^	Ca^2+^	Mg^2+^	Cl^−^	HCO_3_^−^	SO_4_^2−^	NO_3_^−^	NH_4_^+^	NO_2_^−^	Fe^2+^	Mn^2+^	Cu^2+^	Al^3+^	Zn^2+^	Hg^2+^	Pb^2+^	As^3+^	Cd^2+^	F^−^
pH	1.000	−0.265	0.011	0.064	0.019	−0.137	−0.203	0.118	−0.065	0.062	0.136	−0.365^*^	−0.211	−0.185	−0.591	0.149	0.518	0.426	−0.017	0.427	−0.118	0.056	0.389
TH		1.000	**0.612**	0.434	**0.677**	**0.772**	**0.919**	0.414	**0.676**	**0.766**	0.272	0.106	−0.205	−0.126	0.415	−0.193	**−0.616**	−0.324	−0.103	−0.374	0.006	0.150	0.044
TDS			1.000	0.788	**0.829**	0.261	**0.708**	**0.768**	**0.677**	**0.683**	0.183	0.194	0.056	−0.127	0.258	−0.044	−0.273	−0.097	−0.145	0.042	0.172	0.376	0.406
Na^+^				1.000	**0.747**	−0.036	**0.575**	**0.911**	**0.707**	**0.616**	−0.052	0.340	0.279	0.146	0.175	−0.021	−0.187	−0.222	−0.092	−0.025	0.301	0.463	0.353
K^+^					1.000	0.228	**0.808**	**0.693**	**0.700**	**0.778**	0.252	0.272	0.127	−0.097	0.301	0.032	−0.305	−0.276	0.004	0.017	0.013	0.571	0.548
Ca^2+^						1.000	**0.529**	0.038	0.253	0.429	0.406	−0.146	**−0.535**	−0.254	0.122	−0.099	−0.317	0.068	−0.137	−0.204	−0.213	−0.074	−0.142
Mg^2+^							1.000	**0.515**	**0.786**	**0.821**	0.233	0.178	−0.054	−0.093	0.402	−0.225	**−0.669**	−0.446	−0.028	−0.370	0.112	0.251	0.156
Cl^−^								1.000	**0.531**	0.491	0.019	0.239	0.075	0.002	0.066	0.031	−0.147	−0.086	−0.094	−0.136	0.253	0.361	0.381
HCO_3_^-^									1.000	**0.666**	−0.041	0.412	0.163	0.242	0.349	−0.273	−0.403	−0.362	0.045	−0.171	0.321	0.245	0.218
SO_4_^2−^										1.000	0.245	−0.068	−0.090	−0.204	0.241	−0.101	−0.489	−0.336	−0.152	−0.074	−0.112	0.419	0.275
NO_3_^-^											1.000	**−0.493**	**−0.465**	**−0.746**	−0.418	**0.682**	0.010	0.251	−0.082	0.232	**−0.703**	0.088	0.081
NH_4_^+^												1.000	**0.668**	**0.683**	**0.545**	−0.345	0.002	−0.354	0.103	−0.098	0.515	0.283	0.208
NO_2_^−^													1.000	**0.549**	0.329	−0.175	0.071	−0.287	−0.087	0.091	0.480	0.311	0.091
Fe^2+^														1.000	0.409	**−0.560**	0.063	−0.218	0.076	−0.105	**0.591**	0.126	0.032
Mn^2+^															1.000	−0.499	−0.494	−0.475	0.097	−0.330	0.359	0.045	−0.046
Cu^2+^																1.000	0.358	0.394	−0.240	0.447	**−0.628**	0.065	0.204
Al^3+^																	1.000	**0.644**	−0.180	**0.630**	−0.172	0.053	0.259
Zn^2+^																		1.000	−0.185	0.330	−0.105	−0.246	0.169
Hg^2+^																			1.000	−0.079	0.138	0.024	0.005
Pb^2+^																				1.000	−0.368	0.374	0.277
As^3+^																					1.000	−0.098	−0.083
Cd^2+^																						1.000	0.488
F^−^																							1.000

Good correlations are represented in bold format.

**Table 3:** Groundwater samples of the study area exceeding the allowable limits prescribed by WHO for drinking purposes.

Parameters	WHO [60]		Percentage of Samples Exceeding
(mg/L)	Desirable Limit	Maximum Allowable Limit	(%)
pH	7–8.5	9.2	Nil
TH	100	500	23.3
TDS	500	1500	16.7
Na	-	200	20.0
K	-	12	40.0
Ca	75	200	Nil
Mg	50	150	Nil
Cl	250	600	13.3
SO_4_^2−^	200	400	Nil
NO_3_^-^	50	-	Nil
NH_4_^+^	1.5	-	3.3
NO_2_^−^	3	-	Nil
Fe	-	0.3	20.0
Mn	-	0.1	30.0
Cu	-	2.0	Nil
Al	-	0.1	Nil
Zn	-	3.0	Nil
Hg	-	0.001	Nil
Pb	-	0.01	Nil
As	-	0.01	6.7
Cd	-	0.003	Nil
Cr	-	0.05	Nil
F	-	1.5	Nil

**Table 4:** Classification of groundwater based on TDS, total hardness (TH), sodium adsorption ration (SAR), soluble-sodium percentage (SSP).

Quality Parameter	Ranges	Categories	Percent of Samples
TDS (mg/L)	<500	Desirable for drinking	13.33
	500–1000	Permissible for drinking	63.33
	1000–3000	Useful for irrigation	23.33
	>3000	Unfit for drinking and irrigation	Nil
Total hardness (mg/L)	<75	Soft	Nil
	75–150	Moderately hard	Nil
	150–300	Hard	30.00
	>300	Very hard	70.00
SAR	<10	Excellent	83.33
	10–18	Good	13.33
	18–26	Doubtful	3.33
	>26	Unsuitable	Nil
SSP	<20	Excellent	6.67
	20–40	Good	40.00
	40–60	Permissible	36.67
	60–80	Doubtful	10.00
	>80	Unsuitable	6.67

## Appendix A

**Table A1:** Chemical characteristics of surface water in the study area.

	Sample		Indicator/mg·L^−1^									
		Type	Na^+^	K^+^	Ca^2+^	Mg^2+^	Cl^−^	HCO_3_^−^	SO_4_^2−^	NO_3_^−^	NH_4_^+^	TDS
3	Huiping Town	Cl-Na	113.0	130	36.8	22.7	203.6	168.4	36.2	0.41	1.24	601.00
11	Wucang Port	Cl-Na	2210.0	125.3	90.1	177.4	3780.0	151.3	179.6	1.18	0.65	6690.00
17	Xiexing Port	Cl-Na	8200.0	350.0	308.0	572.0	13,800.0	87.9	844.4	0.73	0.34	30,000.00
30	Lvsi Town	HCO_3_ -Ca·Mg	41.8	9.1	37.8	11.3	71.2	133.0	35.5	1.11	0.59	324.00
3832	Sanhe Port	HCO_3_ -Ca·Mg	33.1	7.7	34.0	10.2	47.9	119.6	31.5	1.38	0.06	273.00
